# Depolarizing American voters: Democrats and Republicans are equally susceptible to false attitude feedback

**DOI:** 10.1371/journal.pone.0226799

**Published:** 2020-02-05

**Authors:** Thomas Strandberg, Jay A. Olson, Lars Hall, Andy Woods, Petter Johansson

**Affiliations:** 1 Lund University Cognitive Science, Lund University, Lund, Sweden; 2 Department of Psychiatry, McGill University, Montreal, Quebec, Canada; 3 StoryFutures, Department of Psychology, Royal Holloway University of London, Egham, Surrey, England, United Kingdom; Mälardalen University, SWEDEN

## Abstract

American politics is becoming increasingly polarized, which biases decision-making and reduces open-minded debate. In two experiments, we demonstrate that despite this polarization, a simple manipulation can make people express and endorse less polarized views about competing political candidates. In Study 1, we approached 136 participants at the first 2016 presidential debate and on the streets of New York City. Participants completed a survey evaluating Hillary Clinton and Donald Trump on various personality traits; 72% gave responses favoring a single candidate. We then covertly manipulated their surveys so that the majority of their responses became moderate instead. Participants only noticed and corrected a few of these manipulations. When asked to explain their responses, 94% accepted the manipulated responses as their own and rationalized this neutral position accordingly, even though they reported more polarized views moments earlier. In Study 2, we replicated the experiment online with a more politically diverse sample of 498 participants. Both Clinton and Trump supporters showed nearly identical rates of acceptance and rationalization of their manipulated-to-neutral positions. These studies demonstrate how false feedback can powerfully shape the expression of political views. More generally, our findings reveal the potential for open-minded discussion even in a fundamentally divided political climate.

## Introduction

The political landscape in the United States is becoming increasingly polarized [1–4]. Studies have shown that this polarization biases political decisions as well as reduces informative and critical thinking. For example, people tend to automatically support policy issues proposed by their own party and reject those coming from the opposition [5]. Even during effortful deliberation, people usually side with their own party’s stance on various issues [6]. Furthermore, polarization strongly correlates with confirmation bias: polarized individuals are more inclined to seek and interpret information to confirm their present ideas about the world [7]. Recent studies have also indicated that people are more susceptible to disinformation and less likely to trust sources that do not fit their agenda [8].

Polarization also extends beyond policy issues into personal relations. The levels of animosity directed towards the opposition have dramatically increased over the past decade. In 2008, about 20% of Democrat supporters and 30% of Republican supporters reported feelings of hatred for their counterparts. In 2016, levels of hatred had risen to about 50% for both parties [1]. Most voters now report that people supporting the opposition anger and even scare them [9–10]. In a telling example, Chen and Rohla [11] found that Thanksgiving dinners in 2016 were 30 to 50 minutes shorter for families consisting of both Democrats and Republicans, compared to same-party families. Across the United States, this meant a loss of up to 34 million hours of cross-partisan Thanksgiving discussions that year, likely contributing to further polarization.

Candidates and campaign strategists leverage this powerful affective dimension of polarization to highlight their personality and leadership abilities [12–13]. This strategy was particularly salient during the 2016 American presidential election. Indeed, the contrast in personality and character between the candidates became a near obsession in both the campaigns and the media [14–15], a pattern likely to repeat in the upcoming election cycle. For example, during the final two presidential debates, the majority of questions that the moderator asked concerned the candidates’ characters—even including questions such as whether it is okay for a president to be “two-faced”. In the aftermath of the election, analysts expressed concerns that this trend of personality over policy would lead to even further polarization and animosity among voters [16–17]. These concerns have also persisted throughout Trump’s presidency, culminating in debate about whether his rhetoric might have contributed to the increase in politically motivated hate crimes [18] and acts of domestic terrorism such as the mail bombs sent to Democratic politicians [19–22]. Given this troublesome situation, attempts have been made to create a civic depolarization movement to promote open-minded attitudes and to make people more accepting of different political views [2, 23–25]. However, to be effective, such a movement would require a firm grasp on the nature of attitude depolarization. Thus, there is a pressing need for research that provides more knowledge about people’s propensity to be more open and flexible in their political reasoning.

One way to experimentally make people consider ideas that are ideologically different from their own is through the *choice blindness paradigm*. Choice blindness is a cognitive phenomenon that occurs when people receive false feedback about a choice they had made, leading them to accept the outcome as their own and confabulate reasons for having made that choice in the first place (see [26] for details). Recently, choice blindness has been applied to the study of attitude change, an area of research that struggles to elucidate the dynamics between the stability and flexibility of attitudes. For example, in Hall, Johansson, and Strandberg [27], participants accepted 60% of the manipulations to a survey on moral dilemmas as their own attitudes. Similar findings have been reported during general elections in both Sweden [28] and Argentina [29]. Hall and colleagues [28] also found that participants not only changed their attitudes on political issues, but their actual voting intention was also affected in the direction of the false feedback (which was not found in [29]). Notably, Strandberg and colleagues [30] found that when participants accepted the manipulations of political attitudes, their attitudes shifted congruently with the false feedback and even persisted one week later.

Choice blindness has proven to be an effective tool for creating situations in which people’s flexibility and openness to different political perspectives can be studied. However, as far as we know, choice blindness has never been applied during an American election on a topic as polarized, salient, and contentious as the character of presidential candidates. Given the need for reconciliation and open-mindedness in American politics [2, 23–25], we aimed to test whether we could depolarize American voters, making them more open in their judgments of competing candidates. A few weeks before the 2016 election, we asked participants to fill out a survey assessing the character traits of presidential candidates Donald Trump and Hillary Clinton. We then covertly shifted their polarized ratings to become more moderate. We hypothesized that participants would fail to notice this manipulation and would instead accept and rationalize the altered position as their own. We also wanted to see whether changes in perceived open-mindedness would generalize to judgments of presidential competency.

## Experiment 1

### Method

#### Participants

Posing as political researchers, we recruited 136 participants in New York during the week of the first 2016 presidential debate, six weeks before the election. A third of the participants (*n* = 41) were recruited at the debate itself (around Hofstra University); the rest were recruited during the same week at parks in New York City (Central Park and Washington Square Park). We excluded data from 14 participants: one was too young to vote, one had trouble seeing the survey, one wished to have his data removed after the debriefing, and the rest had errors in the experimental procedure. After exclusions, 122 participants remained in the final sample (87 females; aged 18 to 42, *M* = 21.7, *SD* = 4.3). Most of them were students (75%), and the others had a wide range of occupations including journalists, professors, farmers, retailers, lawyers, and film makers. Based on a voting intention question at the end of the experiment, 89% said they planned to vote for Clinton, 3% for Trump, and 8% for a third party. The study was approved by the Lund University Ethics Board, D.nr. 2016–1046. The design and analysis were pre-registered online (see https://osf.io/gzymp); the confirmatory tests are explicitly labelled as such throughout. There was one deviation from the pre-registration: we had initially intended to exclude participants who began with more moderate views, but after analysis we decided to keep them and focus on another set of interesting yet exploratory results. This change did not affect any of the confirmatory hypothesis outcomes.

#### Materials and procedure

**Fig 1:**
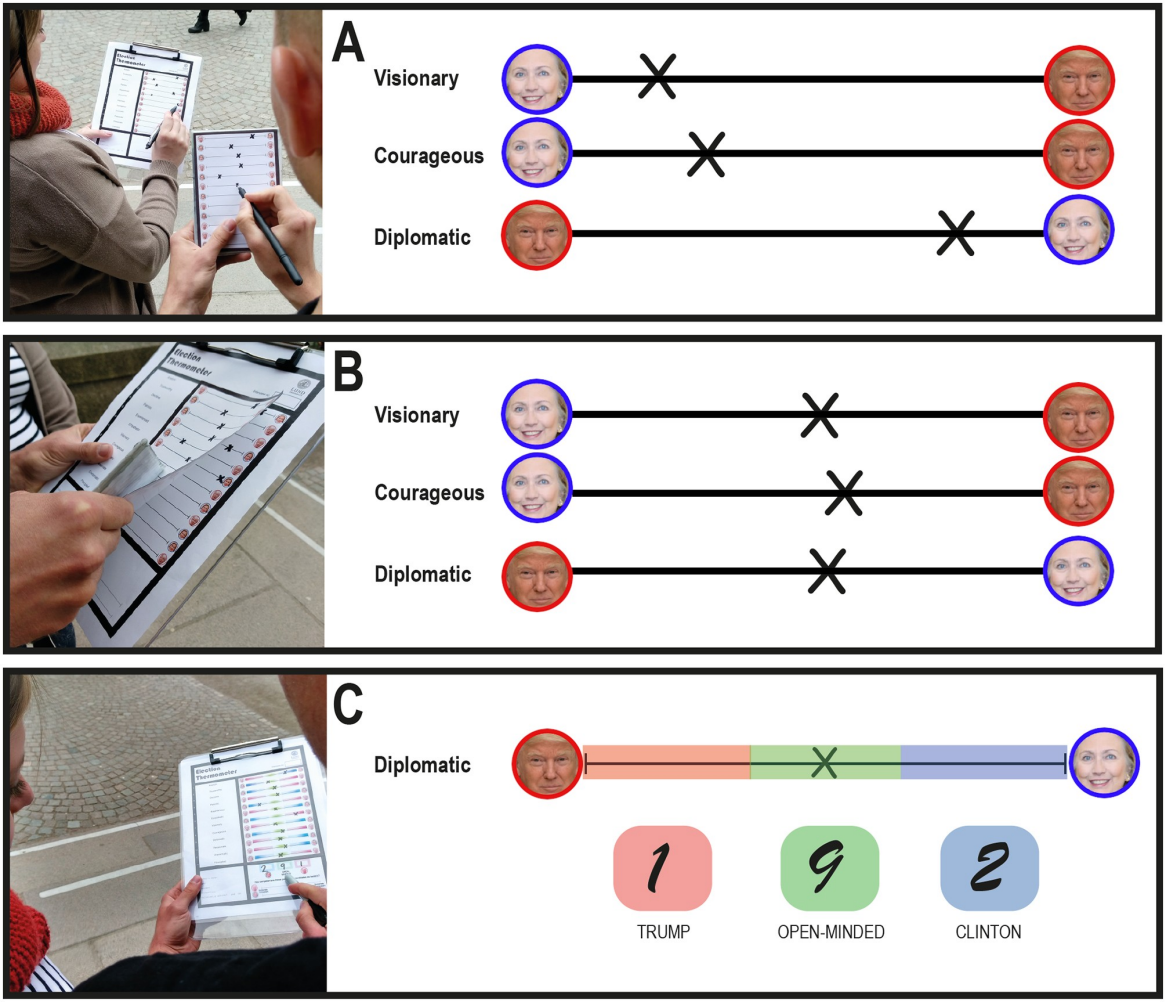
Paper survey. Participants filled out a paper survey rating Hillary Clinton and Donald Trump on 12 leadership traits, such as *courageous* and *diplomatic*. In the experimental group, while participants rated the candidates, we discreetly looked at their ratings and filled out an identical slip of paper with the majority of their polarized ratings shifted closer to the midpoint **(A)**. When the participants finished the survey, we briefly took it and covertly pasted our paper slip with the manipulated moderate responses on top of the participants’ original responses **(B)**. We then asked the participants to explain some of their (manipulated) ratings. Next, we overlaid a transparent sheet that categorized their ratings into: favoring Trump, favoring Clinton, or “open-minded” (i.e., neutral). Together with the participants, we tallied their ratings and asked them to explain their overall score. All participants in the experimental group now had a primarily open-minded score **(C)**. The participants in the control group did not receive any manipulations and instead explained their own original score. (Politician photographs from Wikimedia Commons).

We designed a political survey to assess the leadership traits of two presidential candidates: Hillary Clinton and Donald Trump. The survey items were chosen based on traits that the public usually deems important in a president [31–32]. Participants rated the candidates on 12 adjectives describing leadership traits: analytic, trustworthy, decisive, patriotic, experienced, empathetic, visionary, courageous, diplomatic, passionate, charismatic, and principled. Each trait on the survey was shown on a visual analog scale with pictures of the candidates at either end-point. We asked participants to rate the candidates on each trait; for example, if they thought Clinton was more analytic, they would mark that scale closer to her, or if they thought Trump was, they would mark it closer to him (Fig 1A). To minimize response bias, we randomized which side of the scale Clinton or Trump appeared on for each item. Overall, the responses had good internal consistency (Cronbach’s *α* = .82, 95% CI [.78, .87]).

Participants were randomly assigned to either the control group (*n* = 53) or the experimental group (*n* = 69). We randomized participants such that the majority would be in the experimental condition, since we were more interested in this group. In the experimental group, our goal was to make it appear as if participants had more moderate views than they initially reported. To accomplish this, while the participants rated the candidates on the 12 leadership items, we discreetly observed their responses. At the same time, we filled out an identical slip of paper with some of their most polarized responses shifted closer to the midpoint of the scales (Fig 1A). When the participants finished the questionnaire, we briefly took it to ostensibly review the responses. At this point, we covertly pasted our paper slip with the manipulated moderate responses on top of the participants’ original responses (Fig 1B), then we handed the questionnaire back to them. It now appeared as if the participants had given primarily moderate responses to the questions. This replacement was inconspicuous and took only a few seconds to complete. In the control group, we performed a similar procedure but without manipulating any of the responses. We then asked participants in the control group to explain the reasoning behind approximately three arbitrary non-manipulated responses; in the experimental group, we asked about three manipulated ones. The experimenter would ask, for example, “Why do you think that Trump is more analytic?”. If the participants hesitated, or behaved as if something were wrong, the experimenter would inform them that they could change their response (operationalized as *correction*) and instead explain their reasoning behind that response. We tape-recorded the reasons participants gave to each of these responses.

Next, we told the participants we would calculate a summary score of their responses using a transparent overlay that segmented the scales into three categories: a clear preference for Trump, a clear preference for Clinton, or “open-minded” in the middle 30% of the scale (Fig 1C). Together with the participants, we tallied their 12 responses into the three categories. Using this segmentation rule, participants received summary feedback that their score had a majority of either Trump, Clinton, or open-minded responses. We then showed the participants their overall score and asked them, “Most of your responses were in the open-minded (or Clinton, or Trump) category–do you know why this would be?” We tape-recorded as participants explained their overall score. (Two participants did not want their voices recorded and were thus excluded from this measure.) Two independent judges later assessed whether participants justified the manipulated position. In particular, the judges rated whether participants provided clear justifications (e.g., “My parents raised me to be open-minded”), versus whether they either rejected the score (e.g., “I don’t think I’m that open-minded”) or did not justify it at all (e.g., “I don’t know”). We conservatively defined justification as occurring only when both judges agreed that the participant justified the score; the judges agreed on 75% of their ratings.

Having discussed their aggregate score, we next asked participants to rate the candidates’ competency (“How competent are these candidates as leaders?”), to see if the manipulation and confabulation would affect these more general attitudes. Here, each candidate had a visual analog scale ranging from “Extremely incompetent” to “Extremely competent”. We then debriefed the participants, asked who they were planning to vote for, and finally asked for consent to use their data.

### Results

#### Correction of the false feedback

In the experimental group, we manipulated an average of 8.53 responses closer to the midpoint of the scale, with 3.55 of these moving from supporting one candidate to being in the open-minded category. We then asked participants to explain approximately 3 (*M* = 3.1, *SD* = 0.49) of these manipulated responses, and they only corrected 12% (95% CI [8%, 17%]) of these. Overall, 28% of the participants corrected one manipulation and only 4% corrected two. None corrected more than two of the discussed responses. The participants who made the corrections said that they had either made an error or changed their mind about the rating. No participants expressed any suspicion that their responses had been manipulated, even when asked after the study if they had noticed anything unusual. Accordingly, the participants accepted the large majority of the manipulated responses as their own. After accepting the manipulated responses, participants often gave elaborate arguments for them. For example, one participant marked his response to the *experienced* item as 94% on the Clinton side of the scale, which we manipulated to a more neutral position closer to the middle of the scale (59%). When asked to explain the latter rating, he said, “I think they’re both experienced in their field. Trump is a really successful businessman … And then, Hillary has had a lot of years [of] practice in office. So I … feel like they both are really experienced.” Another participant originally rated *diplomatic* as 73% on the Clinton side, which we changed to more neutral (57%). She stated, “Hillary has been in the political scene for a very long time, but I think also Trump has a diplomatic aspect to him just because he is very passionate … about the country.” Participants thus offered arguments for moderate positions even though they had originally reported more polarized opinions just moments earlier.

#### Manipulation, acceptance, and justification of the aggregate survey score

**Fig 2:**
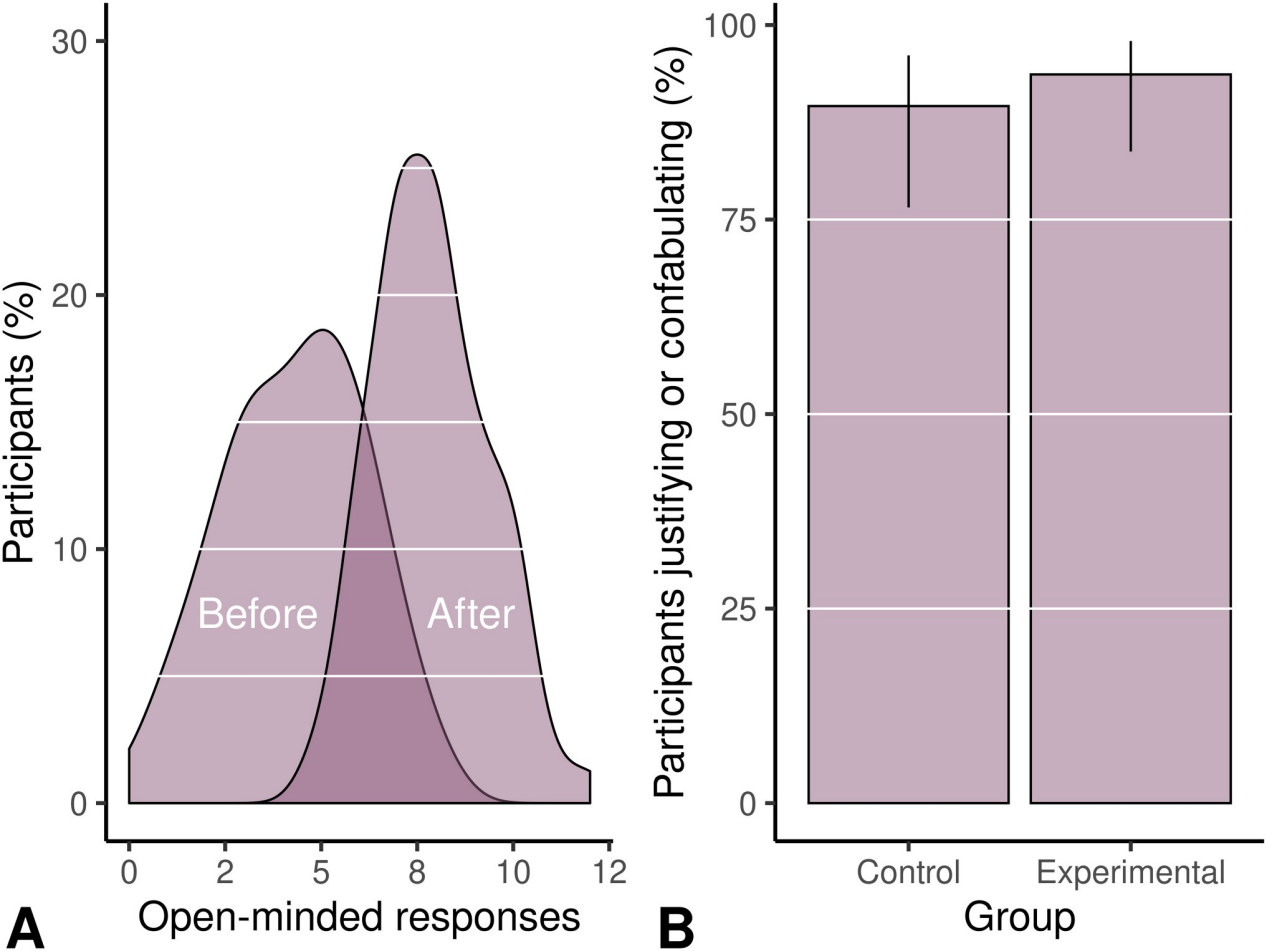
Frequency of “open-minded” responses and justification rates. The feedback made it appear as if participants had provided more open-minded responses (A); they then explained the reasons behind their original views or the manipulated ones (B).

Our false feedback made it appear as if participants were overall less polarized. In the experimental group, participants originally had an average of 4.32 (95% CI [3.88, 4.75]) neutral responses out of 12; after the manipulation and correction phase, the participants were given the feedback that they had 7.87 [7.52, 8.20] of them (Fig 2A). Looking only at participants that had an overall polarized score (i.e. a majority of responses favoring a single candidate), they had 3.20 [2.79, 3.59] neutral responses before the manipulation and 7.27 [6.70, 7.77] after it. Originally, 25% [15%, 37%] of participants in the experimental group had a majority of neutral responses, and the false feedback suggested that almost all of them (97%) did. The control group experienced no manipulation, and 30% [19%, 45%] of them had primarily neutral responses. As expected, in the control group, the large majority of participants (90% [77%, 96%]) verbally justified their own original views, whether neutral or polarized (Fig 2B).

Surprisingly, in the manipulation group, a similar number of participants justified their *manipulated* views which they did not hold moments earlier (94% [84%, 98%]). For example, one participant heavily favored Trump; after the false feedback about open-mindedness, he claimed, “I feel like Clinton and Trump are both in the middle and I don’t really stand for either of them.” Another participant who initially favored Clinton stated, “I guess I fall somewhere in the middle–I’d like to think I’m a little moderate. … I think at this point it’s important to be open-minded.” Others discussed balancing the strengths and weaknesses of both candidates: “In terms of being decisive, Trump is more exact and confident in his decisions, so that could be viewed as being decisive. But then Hillary has a track record in which she’s changed her mind about a lot of issues, but that’s kind of like her educating herself and having developed thought. So that’s two different ways of looking at it.”

#### Competency rating

At the end of the experiment, we asked participants to evaluate both candidates’ competence as leaders. The average absolute difference in competency ratings between the candidates was 48.37 [39.39, 56.04] in the control group and 53.45 [47.03, 59.82] in the experimental group. Confirmatory tests showed that these differences did not vary by group (*t*(120) = 0.95, *p* = .345), nor did individual ratings for Clinton (Wilcoxon-Mann-Whitney *Z* = -.400, *p* = .691) or Trump (*Z* = .599, *p* = .550). This indicates that while participants in the experimental group often endorsed and rationalized their seeming open-mindedness, the manipulation did not affect their overall candidate judgments.

### Summary of Experiment 1

We found that participants rarely detected when their evaluations of the two presidential candidates had been manipulated into a more “open-minded” position. Instead, they accepted the altered responses as their own and offered unequivocal justifications for them. In the end, this made them endorse a substantially more neutral position compared to their original score. This finding builds upon and supports previous studies exploring false feedback and political attitudes [28–30]. However, choice blindness had never been applied to study depolarization of candidate evaluations during an American election.

## Experiment 2

One major caveat of Experiment 1 is that, due to the location of the first presidential debate in New York, our sample was heavily skewed towards the Democratic Party. Looking at the overall tally of the responses for all participants, 85% had more responses favoring Clinton and only 11% favored Trump. This was further reflected in the general competency rating: 89% of participants thought Clinton was more competent and planned to vote for her. Typically, we would not be concerned with this limitation, as we have no prior reason to expect that Republican supporters would behave differently from Democrats. Choice blindness studies generally have given few indications that individual differences are key to explaining the effect. However, two factors may make the present situation unique. First, the stakes are considerably higher, as research on political attitudes is often weaponized and wielded in the public debate on polarization. Second, and more important, studies on potential individual differences between liberals and conservatives have become a hotbed of activity, with many contentious results and speculative interpretations. A choice blindness study with participants from the full political spectrum could provide a valuable contribution to this debate. Thus, we decided to run a second experiment with a larger and more representative sample.

In the ongoing chase for dissimilarities in personality and cognitive processing between liberals and conservatives, there is some evidence that personality might differ between them. In the popular Big Five personality inventory, liberals score higher on openness to experience whereas conservatives score higher on conscientiousness [33–34]. When it comes to universal values, people on the left tend to value universalism and benevolence, whereas people on the right tend to value achievement and tradition [35]. Researchers have also underlined differences in moral reasoning; liberals tend to favor particular foundations (e.g., harm/care, fairness/reciprocity) whereas conservatives put more emphasis on others (e.g., authority/respect [36–37]). Several studies have also found differences in thinking styles: conservatives have been seen as more intuitive and heuristic, whereas liberals have been seen as more analytic and systematic (e.g. [38–39]). In line with this, two studies found indications that “bullshit receptivity”—the propensity to believe statements independent of their truth—was higher for conservatives [40–41].

On the other hand, it is unclear how these findings translate to the realm of polarization, as studies of political cognitive processing seem to indicate that conservatives and liberals are similarly sensitive to various biases. For example, Frimer, Skitka and Motyl [42] found that the opposing camps were equally averse to statements that did not support their political position. Even when participants had a chance to earn money by simply reading counter-ideological statements, about two thirds of both liberals and conservatives declined to do so, indicating that there is a considerable mental “cost” involved in exposing oneself to opposing information and arguments. Furthermore, in a meta-analysis of 43 studies investigating various biases, the researchers found almost identical levels of partisan bias and confirmation bias for both liberals and conservatives [43]. Similarly, the propensity to believe fake news has also been found to rely on factors such as analytic thinking and prior exposure, rather than partisanship [44–45].

It remains unclear whether liberals and conservatives would differ on a novel decision measure like choice blindness, which involves a combination of false feedback and potential confabulation not used in any of the studies previously discussed. Susceptibility to false feedback has not systematically been linked to ideology, and political choice blindness studies conducted in Sweden and Argentina have yielded mixed results (see [27–30] for details). However, the two-party electoral system in the United States, fueled by higher levels of polarization, is an ideal domain to explore this research question. Thus, in Experiment 2, we aimed to replicate Experiment 1 testing both liberals and conservatives. To accomplish this, we designed an online version of the first experiment in order to reach a larger and more representative population.

### Method

#### Participants

Experiment 2 took place a few days before the general election being held on November 8, 2016. Participants were 498 (60% male) American citizens with an average age of 31.1 years (*SD* = 10.1). They were recruited through the online survey platform Prolific Academic [46] and asked to participate in a political survey. Participants were randomly assigned to either the experimental condition (*n* = 405) or the control condition (*n* = 93). The experiments ran on the software Xperiment version 2 [47]. Participants received $2.50 USD as compensation. The study was approved by the Lund University Ethics Board, D.nr. 2016–1046.

#### Materials and procedure

**Fig 3:**
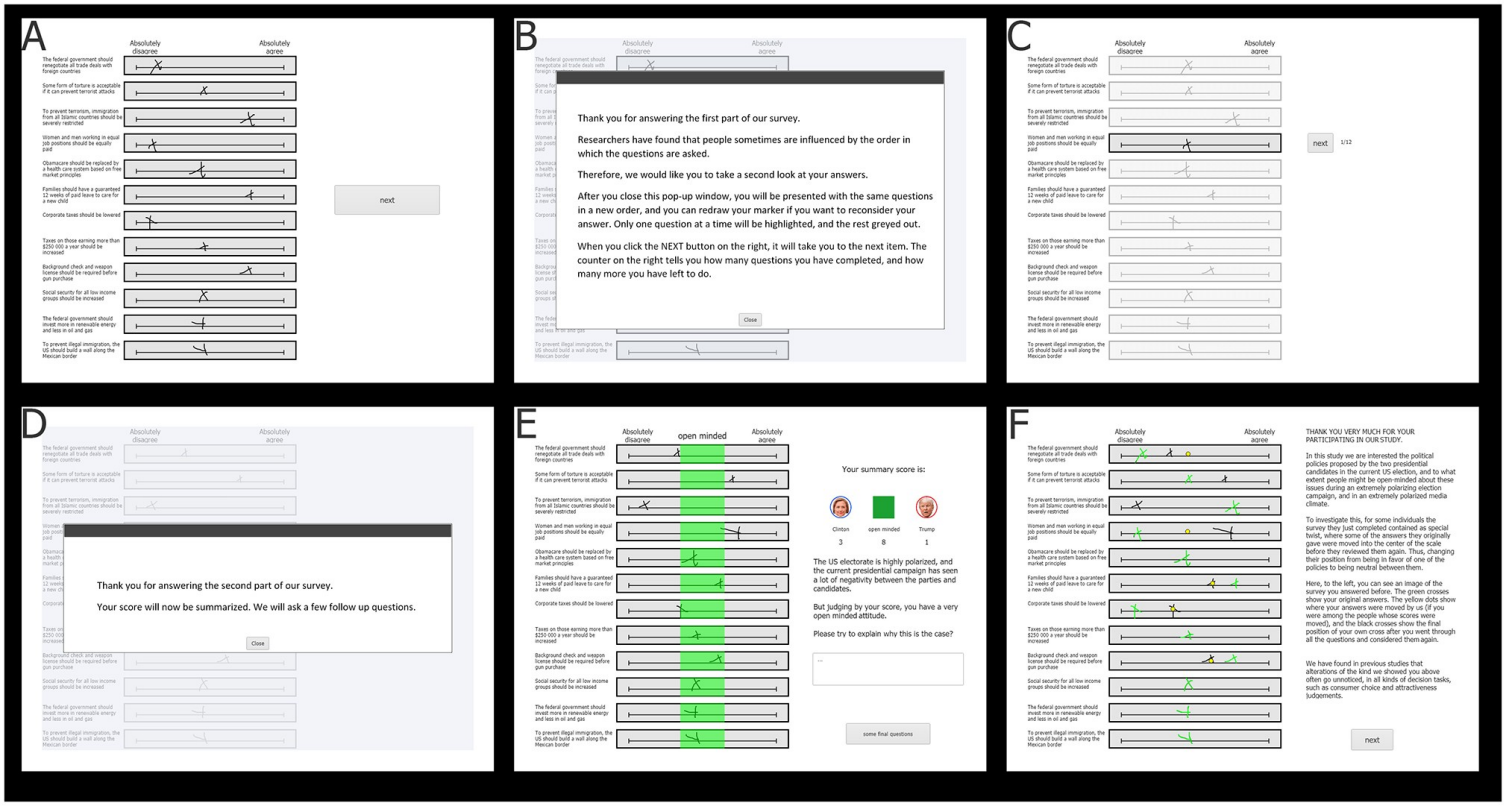
Online survey. Participants rated Hillary Clinton and Donald Trump on 12 leadership traits **(A)**. They were then instructed to look over their responses and were told that they could change any response by drawing a new one **(B)**. The items were presented one by one with the rest blurred. Participants in the experimental group received five manipulations that moved each response to a more moderate position **(C)**. Participants were then told that their score would be summarized **(D)**. They received a score showing how many of their ratings were in each of the three categories (Clinton, Trump, and open-minded). They were also told their degree of open-mindedness based on the number of their responses in the green middle segment and were asked to explain this in text **(E)**. They then rated their overall preference for the candidates **(F)**.

Experiment 2 followed the same general design and procedure as Experiment 1. The participants completed a 12-item survey and were given a chance to change their responses. They then received a summary score giving them feedback about their level of open-mindedness. The survey consisted of the same leadership traits as used in Experiment 1 (e.g., analytic, trustworthy). At the start, all items were presented as a randomized list on the same page, with continuous scales ranging between Clinton and Trump (Fig 3). Rather than using a pen and paper as in Experiment 1, the participants used their mouse to draw an ‘X’ on the scale where it best represented their attitude towards each item. After the participants had answered all 12 items, they received the following cover story and instructions: “Researchers have found that people sometimes are influenced by the order in which the questions are asked. Therefore, we would like you to take a second look at your answers”. They were then presented with the items and their responses again, but in a different order, and asked to verify or change their previous responses. They were informed that they could change any response by clicking ‘edit’ and drawing a new ‘X’. The items were presented one at a time, with the other items blurred.

All participants in the experimental condition were given false feedback regarding 5 of their 12 responses. The manipulation mechanism was as follows: select the first five responses at the extremes of the scales (i.e. between 0% and 35% or 65% and 100%), and move them to a random position within the middle 30%. Should a participant have fewer than five responses outside of the middle 30%, the items farthest from the midpoint would be moved closer (by a random amount) towards the midpoint. Thus, all participants received five manipulations shifting their original responses closer to a more open-minded position.

As in Experiment 1, participants then received a summary score showing the list of all 12 items as well as their responses and their associated categories (i.e. Trump, open-minded, Clinton). The participants’ degree of open-mindedness was also described in text: “judging by your score, you have a…” followed by: “…somewhat open-minded attitude” (0–2 open-minded responses), “…open-minded attitude” (3–6), “…very open-minded attitude” (7–10), or “…extremely open-minded attitude” (11–12). Participants were then prompted to type an explanation describing their degree of open-mindedness. The last part of the experiment consisted of the question, “How would you compare the two candidates?”, with a continuous scale between Clinton and Trump. Finally, the participants were debriefed and asked for their data to be used for research purposes.

### Results

#### Analysis

In Experiment 2, we did not explicitly ask participants who they were going to vote for in the election. Instead, we based their candidate support on their original aggregate survey score and categorized the participants as either Clinton supporters or Trump supporters using a simple majority rule. Participants with a majority of responses favoring Clinton were categorized as Clinton supporters, participants with a majority favoring Trump were Trump supporters, and participants with a majority of “open-minded” responses were categorized as open-minded. Following this rule, the sample consisted of 234 Clinton supporters, 75 Trump supporters, 147 open-minded, and 42 ties in which no category has a majority. To further corroborate this classification, we compared how Clinton and Trump supporters answered the favorability question (“How would you compare the two candidates?”), with a scale ranging from Trump (0) to Clinton (100). As expected, the two groups differed in their ratings (Clinton supporters: *M* = 86.92 [84.84, 88.94], Trump supporters: *M* = 18.93 [13.94, 24.38]) indicating that this is a valid categorization of the participants’ candidate preference. Similar to Experiment 1, two independent judges categorized participants’ explanations based on whether they justified or rejected their ostensible open-mindedness. The judges agreed on 62% of their ratings, which was lower than in Experiment 1. This lower reliability was likely due to the poorer quality of responses; judges were making their decisions based on short phrases or sentences, while in Experiment 1 they had audio recordings lasting several minutes to provide more context.

#### Correction of the false feedback

We manipulated five responses for each participant to a more neutral position, and the participants were confronted with all manipulations. Of these, 41% of the total 2025 manipulations were corrected. On average, participants corrected 2.06 [1.85, 2.24] manipulations. In total, 154 participants made no corrections, and 71 corrected all of the manipulations. When we compare the correction rates of Clinton and Trump supporters, we find no difference: Trump supporters corrected 2.36 [1.87, 2.88] items on average while Clinton supporters corrected 2.32 [2.02, 2.60] (Wilcoxon-Mann-Whitney *Z* = .18, *p* = .861). Participants who began with a majority of responses in the open-minded category had a lower correction rate (1.47 [1.15, 1.77]) compared to participants favoring a specific candidate (Wilcoxon-Mann-Whitney *Z* = 3.66, *p* < .001). However, this is probably best explained by the fact that the manipulation seemed less extreme since they were already more neutral.

#### Manipulation, acceptance, and justification of the aggregate survey score

**Fig 4:**
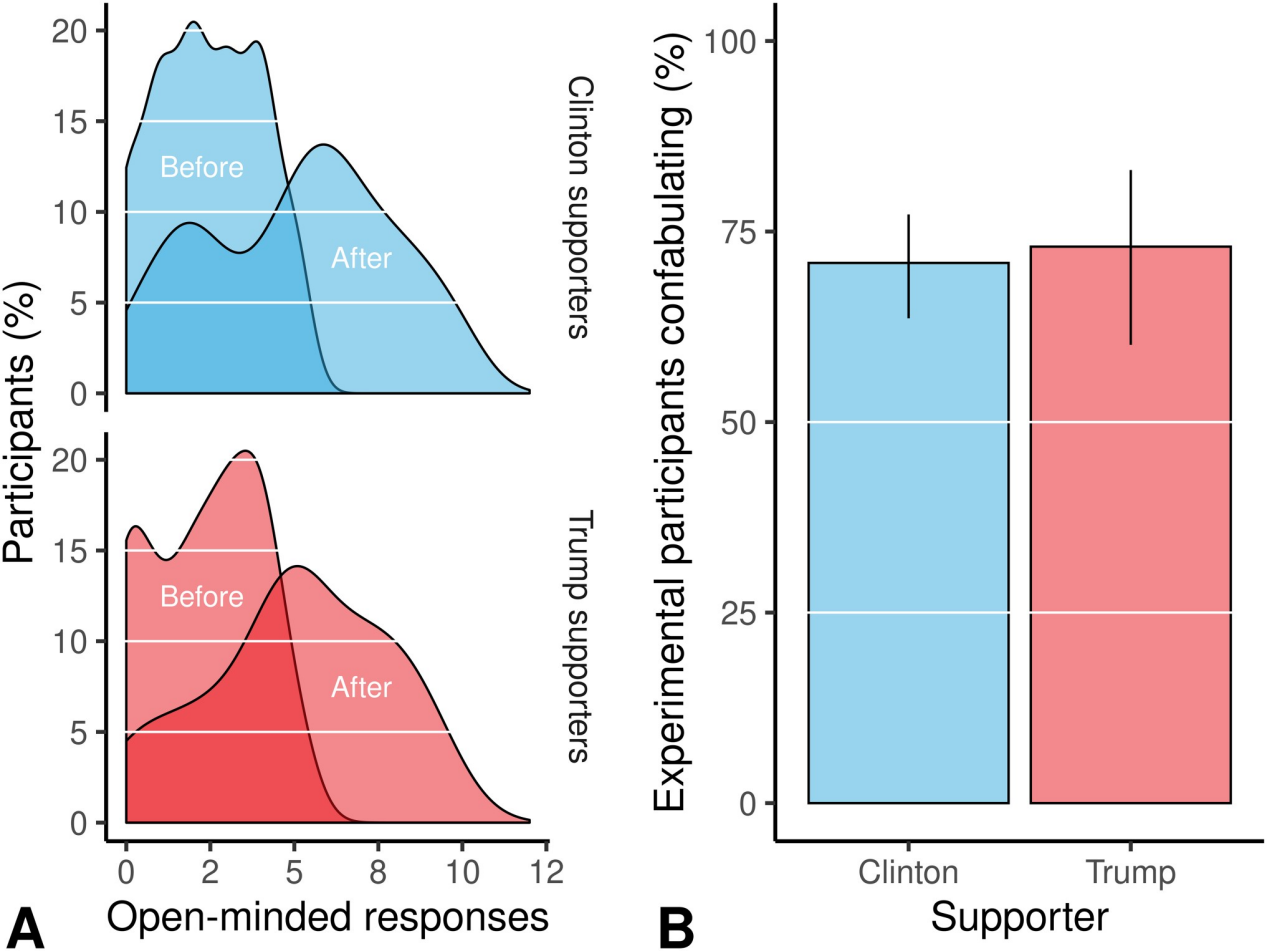
Frequency of “open-minded” responses and confabulation rates in the experimental group. As in Experiment 1, the manipulation made it appear as if the participants had provided more open-minded responses (A); they then explained the reasons behind their original views or the manipulated ones (B). We saw similar rates for both Clinton and Trump supporters.

Originally, the participants had on average 4.06 [3.80, 4.33] neutral responses; after being exposed to and correcting the manipulations, they had 6.71 [6.37, 7.04] neutral responses (Fig 4A). Importantly, both Clinton (2.43 [2.21, 2.63]) and Trump supporters (2.33 [1.95, 2.74]) began with the same number of neutral responses. After the manipulation and corrections, this amount had doubled (Clinton supporters: *M* = 5.07 [4.65, 5.47]; Trump supporters: *M* = 5.07 [4.44, 5.71]). As a result of this, when participants received a description at the end about their level of open-mindedness, they were most often told “you have an open-minded attitude” (i.e. between 4 and 7 open-minded responses). They were then given the opportunity to explain their open-mindedness in text and these were analyzed by independent judges. Overall, the confabulation rates in the experimental group were high (71% [64%, 78%] for Clinton supporters and 73% [60%, 83%] for Trump supporters; Fig 4B), meaning that both Clinton and Trump supporters justified their apparent open-mindedness. There was no difference in their degree of justification (χ^2^(1, *N* = 245) = 0.03, *p* = .872).

#### Favorability rating

In Experiment 1, the open-mindedness manipulation did not influence participants’ overall competency ratings. In Experiment 2, we instead asked participants to rate their favorability: “How would you compare the two candidates?” Again, we saw no differences between the control group (*M* = 69.40 [63.32, 75.27]) and the experimental group (*M* = 64.08 [61.06, 67.03]; Wilcoxon-Mann-Whitney *Z* = 1.43, *p* = .154), and in both groups Clinton supporters favored Clinton (*M* = 86.93 [84.77, 88.80]) whereas Trump supporters favored Trump (*M* = 18.93 [13.93, 24.71]). This shows that even though participants in the experimental group endorsed and justified their apparent open-mindedness, Trump supporters still rated Trump as more favorable, and Clinton supporters rated Clinton as more favorable. As in Experiment 1, changes in individual character evaluations do not necessarily influence overall favorability.

## Discussion

There is an ongoing quest to create a less polarized and more open-minded political climate in the United States [2, 23–25]. We believe this to be an important effort for several reasons. Studies show that polarization can bias information processing and decision making in detrimental ways [5–6, 48]. As a result, it often leads to fear, anger, and animosity towards the opposition [1, 9–10]. Polarization is also associated with dogmatic intolerance, which in turn increases the propensity to behave antisocially and to deny free speech [49]. Furthermore, polarization erodes central parts of civic society, such as trust in the government and media [50]. However, for a depolarization movement to be effective, we need to advance our theories on political attitude change and better understand the mechanisms underlying depolarization.

To contribute to this effort, we tested the choice blindness paradigm [26] with American voters just before the 2016 American general election. Our aim was to investigate whether participants could become less polarized in their political views. Study 1 was conducted during the week of the first presidential debate; Study 2 was conducted online with a larger and more representative sample. Participants responded to a survey comparing Hillary Clinton and Donald Trump on various leadership traits. In both studies, the participants in our sample were clearly polarized when entering the study. Participants that favored either of the candidates had on average only 2 to 3 “open-minded” responses out of 12, defined by a response in the middle 30% of the visual analog scales. Participants then received false feedback about their responses: we nearly doubled the number of items that participants had in the open-minded category. Only a few of these manipulations were detected and corrected, which resulted in an overall score that made it appear as if the participants were more open-minded in their views towards the candidates. When asked to explain their score, the great majority of the participants accepted and justified their apparent open-mindedness, even though they had reported more polarized views moments earlier.

### Supporters of Clinton and Trump are similarly susceptible to false feedback

In Experiment 2, both Clinton and Trump supporters behaved similarly on the experimental measures: they had similar correction rates to the choice blindness manipulations and justified their open-minded score to similar degrees. This is the first study we are aware of that demonstrates that liberals and conservatives are equally susceptible to false feedback about their own attitudes. Given previous findings that acceptance and justification of false survey feedback can lead to lasting changes in political attitudes [30], we see the lack of difference between Trump supporters and Clinton supporters as contributing to the ongoing research on the psychology of ideology. So far, this line of research indicates that liberals and conservatives are different in some aspects, such as personality [33], values [35–36], and thinking styles [38–39]. However, they are both similarly susceptible to cognitive biases [42–43]. Our findings show that choice blindness applies equally to conservatives and liberals. More generally, choice blindness offers a useful tool to test how liberals and conservatives reason—or rationalize—when presented with false information.

### Choice blindness as a method to study depolarization

The current study was not intended as a practical method to influence voters but rather as a novel investigation of experimental depolarization in the political domain. We find that giving people false feedback can be an effective way to, at least momentarily, make them perceive themselves as more open towards competing candidates. This shows that even deeply held beliefs depend on situational factors and can be flexible under certain circumstances. From a theoretical perspective, we believe that participants interpret their own behavior—in this case their survey responses—and infer the reasons behind these responses [51–54]. Choice blindness could therefore be useful to study the depolarization of extreme views. For example, we could measure how susceptibility to choice blindness and confabulation are affected by the direction of the manipulation, such as going from polarized to moderate, or vice versa. This could help us understand whether being moderate or undecided is a distinct pole of its own. If so, we could explore whether these moderate views are more or less susceptible to false information. Here, the framing of moderate views may play an important role. In our studies, participants received *positive* false feedback about their survey responses. Instead of suggesting to people that they are open-minded, we might have found different results if participants had been told that they were “wishy-washy”, “flip-flopping”, “uncertain”, “centrist”, or even “moderate”. Future work could examine how participants behave when they are given false *negative* or more neutral feedback as well.

The effectiveness of choice blindness in the political domain distinguishes it from many other forms of persuasion, such as perspective-taking [55–56]. In a recent study, Catapano and colleagues [57] found that such methods are less effective for deep-seated attitudes, such as those relating to politics. In fact, imagining the perspectives of out-group members can even backfire and hinder subsequent attitude change. This could partially be explained by the fact that in those paradigms, participants are fully aware that the perspective they consider is not their own and that the arguments they express are hypothetical. In choice blindness experiments, however, participants often believe that the response they are asked to explain reflects their own true attitude.

### Limitations and future studies

In Experiment 1, only 12% of all manipulations were corrected, but in Experiment 2, 41% of them were. The reasons behind this difference are difficult to isolate given the variation in design between the two studies (such as the number of manipulations, the instructions for revisiting their responses, and verbal versus written explanations). One potential explanation is the plausibility of the manipulation. In Experiment 1, the manipulations were performed using a magic trick, which is extremely improbable in the context of a typical political opinion survey. Likely none of the participants had ever filled out a pen-and-paper survey that changed seconds later. Thus, if the participants lack perfect access to their own attitudes (or if political attitudes are not stored for us to access; [58–59]), then the manipulated survey responses ought to function as a prime source of evidence about their own attitudes [51–52]. The (presumably non-conscious) inference may look something like: “I wrote these responses, so either they must be my true attitudes, or else I made several large errors”. So, if people see themselves as competent at answering a simple questionnaire, making a series of large errors would seem less plausible. In contrast, in Experiment 2, even though we attempted to replicate the general procedure of the original trick, participants were faced with a far less magical procedure. People are familiar with malfunctioning computer programs and websites, and thus our participants would have had little difficulty in concluding that there may have simply been a software error when saving their responses that needs correcting.

Another explanation might be the difference between verbally explaining versus silently revising the manipulations. While participants in Experiment 2 were also confronted with the manipulations, they did not have to engage in the mental task of having to recall or generate arguments for them. On the face of it, one might expect this additional reasoning process to generate more corrections, presumably by helping participants think more deeply about the issue and discovering that they do not agree with the manipulated position. However, if deliberation serves not as attitudinal fact-checking but as a way for participants to further commit to and defend their own ostensible attitudes, the reasoning process might lead to fewer corrections [53–54]. A third explanation could be simply that Experiment 2 was conducted closer to the election compared to Experiment 1, and that a larger proportion of the participants in Experiment 2 had firmly decided who they would vote for. Finally, it could also have been that the cover story in Experiment 2—telling participants to check their responses in case they had been affected by presentation order—may have primed participants be more attentive and to search for inconsistencies.

Prior to the current study, choice blindness had only been used to study what might be called “repolarization”—for example by shifting people from agreeing to disagreeing with a statement. Here, for the first time, we show that it is possible to use the same methodology to depolarize people, by making them adopt the idea that they are more “open-minded”.

In future studies, we could also explore more global attitude shifts. In the two experiments presented here, the manipulations did not influence the candidate competency/favorability ratings. Had this been found, it would have been a unique case of attitude generalization where manipulation on some character judgments would bleed over and affect another more general trait. Perhaps political competency is judged somewhat independently of the specific traits in our survey.

## Conclusion

Our findings corroborate a recent large-scale analysis of survey data with answers from 140 000 people across over 60 countries [60]. The researchers found that people across the political spectrum were more similar than they were different on several moral and political attitudes. We share their conclusion that similarities between the attitudes of people and groups tend to be overlooked, suggesting that the “us versus them” dichotomy is a prevalent but perhaps exaggerated narrative. We hope our findings can be used to simulate polarizing societal forces and thus contribute to the search for an effective remedy sought by the political depolarization movements [2, 23–25]. Our study reveals that American voters at either end of the political spectrum are willing to endorse more open views about both candidates with surprisingly little intervention. Here, suggesting to people that they are more open-minded removed their political blinders and nudged them to consider and argue for more moderate views. These results offer hope in a divided political climate: even polarized people can become—at least momentarily—open to opposing views.
